# Twenty years of successful academic outreach at Núcleo de Medicina Tropical (NACE-NUMETROP/USP) in Santarém, Pará

**DOI:** 10.1590/0037-8682-0404-2020

**Published:** 2021-03-22

**Authors:** Renato do Carmo Said, João Guilherme Pontes Lima Assy, Kamila Vieira Silva, Alisson dos Santos Brandão, Olívia Campos Pinheiro, Helena Rangel Esper, Anna Luiza de Fátima Pinho Lins Gryschek, Maria Rita Bertolozzi, Valdir Sabbaga Amato, Marcos Boulos, Aluísio Augusto Cotrim Segurado, Ronaldo César Borges Gryschek, Francisco Oscar de Siqueira França, NUMETROP Supervisors Group

**Affiliations:** 1 Universidade de São Paulo, Faculdade de Medicina, Núcleo de Medicina Tropical, Departamento de Moléstias Infecciosas e Parasitárias, São Paulo, SP, Brasil.; 2 Secretaria Municipal de Saúde de Santarém, Hospital Municipal de Santarém, Santarém, PA, Brasil.; 3 Universidade de São Paulo, Escola de Enfermagem, Departamento de Enfermagem em Saúde Coletiva, São Paulo, SP, Brasil.; 4 Universidade de São Paulo, Faculdade de Medicina, Hospital das Clínicas, Laboratório de Protozoologia, Bacteriologia e Resistência Antimicrobiana (LIM49-IMT2), São Paulo, SP, Brasil.; 5 Universidade de São Paulo, Faculdade de Medicina, Hospital das Clínicas, Laboratório de Imunopatologia da Esquistossomose e outras Parasitoses (LIM06-IMT2), São Paulo, SP, Brasil.; 6 Universidade de São Paulo, Faculdade de Medicina, Hospital das Clínicas, Laboratório de Investigação Médica em Imunologia (LIM48-IMT2), São Paulo, SP, Brasil.

**Dear Editor:**

It is the 20^th^ anniversary of Núcleo de Medicina Tropical (NACE-NUMETROP), an outreach center affiliated to the Department of Infectious and Parasitic Diseases of Faculdade de Medicina da Universidade de São Paulo (DMIP-FMUSP), located in the city of Santarém, Pará and founded in 2000 in outposts in the heart of the Brazilian Amazon in response to a request from the municipal health secretariat (SEMSA). At that time, local authorities required technical support to investigate patients with acute febrile jaundice living in riverside communities. Prof. Marcos Boulos invited Dr. Alexandre Rocha Santos Padilha, then a resident in infectious diseases at USP, to assist in this investigation, resulting in clarifying this public health emergency as an outbreak of leptospirosis. After this successful operation, a joint effort was undertaken by municipal (Dr. Valter Pinheiro Sinimbú -SEMSA) and state (Dr. Bernardo da Silva Cardoso - Department of Endemic Diseases Control, Pará State Secretariat of Public Health - SESPA) health officers to launch a long-standing partnership between local health authorities and the USP. An agreement consolidated the initiative and Dr. Padilha was hired by SEMSA supervise the new Nucleus, to shoulder the responsibility of providing medical care and training local health professionals in the field of Tropical Diseases. Dr. Padilha later became the Brazilian Minister of Health (2011-2014).

Since then, NUMETROP has never ceased to grow. The number of medical supervisors, attending residents and researchers; the breadth of its partnerships; and the scope of activities has been progressively scaled up. Until February 2020, 22 supervisors and more than 400 residents from dozens of universities have worked at the center.

**FIGURE 1:**
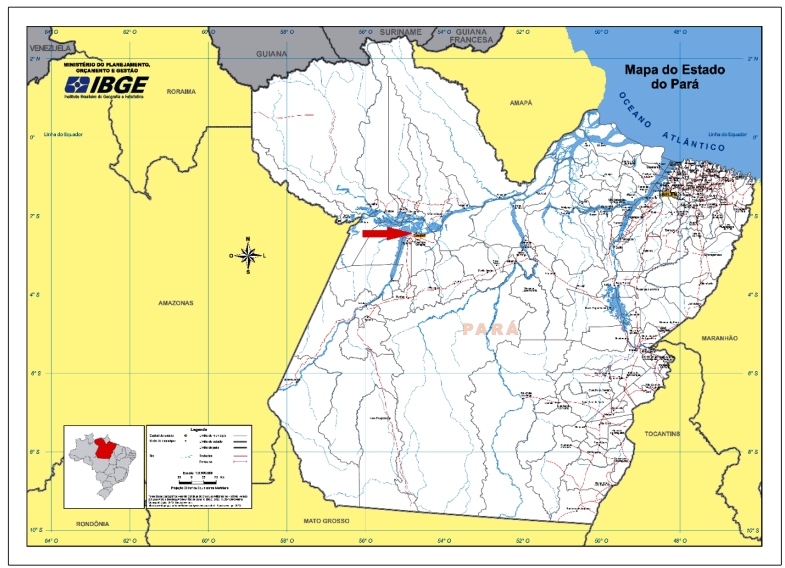
Santarém city - Pará State - Brazil - https://biblioteca.ibge.gov.br/visualizacao/livros/liv64824_mapa_pa.pdf.

Santarém, located in the Lower Amazon region, has an estimated population of 302,667 inhabitants^1^. It lies at the junction of the Tapajós and Amazonas rivers, equidistant from the two largest urban centers in the Brazilian Amazon, Manaus and Belém (Figure 1). Currently, 70% of the population lives in urban areas, and the remaining lives in rural areas in hard-to-reach settlements (countryside, forest, and riverside communities). The city hosts SESPA’s 9th Regional Health Center and plays an important role in the Unified Health System (SUS- Sistema Único de Saúde) structure: a referral destination for patients with medium and high complexity healthcare needs from the 20 surrounding municipalities in the Tapajós and Baixo Amazonas regions. In 2018, this territory had a population estimate of 988,871 inhabitants, spread out over an area of 505,458 km², which is larger than that of Italy, England, the Netherlands, and Belgium put together^1^. Santarém has had an accelerated yet poorly organized urbanization, which, combined with the advances of the national agricultural frontier, has led to intense human-driven deforestation, ecological imbalance, and consequent increases in the incidences of tropical and infectious diseases. Since its creation, NUMETROP has continuously carried out healthcare, teaching, and research activities in the city, mainly at Santarém Municipal Hospital (HMS). This 200-bed setting is a secondary reference hospital for the entire western region of Pará. In this facility, NUMETROP Medical Supervisors conduct in-patient care at the Infectious Disease ward and emergency room and act as consultants for patients admitted to other wards. In addition, they manage out-patients at the General Infectology Clinic. 

Furthermore, NUMETROP conducts healthcare activities in other services affiliated with SEMSA, such as the Citizen Reference Center/ Human immunodeficiency virus (HIV) Testing and Counseling Center and Specialized Outpatient Clinic (CTA/SAE), where patients with HIV/Aids, other sexually transmitted infections, and viral hepatitis are followed up. In addition, the work performed at the SESPA Zoonosis Control Center, (reference unit for patients with cutaneous leishmaniasis), as well as at the primary care center at Alter do Chão^2^ focused on rural and urban healthcare in the neighboring district, are also noteworthy. Within the scope of Epidemiological Surveillance, NUMETROP investigates outbreaks of infectious diseases such as the episode of orally transmitted acute Chagas disease^3^.

Academic cooperation is another strategic initiative taken by the nucleus. In agreement with the Federal University of Western Pará (UFOPA), NUMETROP staff provide healthcare to riverside communities on the Abaré hospital boat. Moreover, an agreement was recently signed with the Santarém Campus of the State University of Pará (UEPA) to support courses and symposia for undergraduate students and local health professionals, addressing locally relevant health issues.

This endeavor has been made possible with the support of many local, regional, national, and international partners, such as SESPA, the Department of Nursing in Collective Health at the University of São Paulo School of Nursing (EEUSP), the Butantan Institute, the Brazilian Society of Tropical Medicine, and the fundamental aid of the Brasilia headquarters of the Pan American Health Organization and the Ministry of Health.

Despite structural difficulties and limited human resources, the coordinators and supervisors of NACE-NUMETROP have supported clinical research activities with emphasis on locally relevant diseases, including those on malaria chemotherapy that led the Ministry of Health to revise the national treatment guidelines^4^. Other outstanding contributions include innovation in the diagnosis of mucocutaneous leishmaniasis^5^ ^,^ ^6^, descriptions of scorpion stings that evolve into acute cerebellar dysfunction^7^, and reports of snakebites and local accidents due to venomous aquatic animals^8^ ^-^ ^10^. Activities of the Citizen Reference Center - CTA/SAE resulted in a better understanding of how the HIV epidemic evolved in the region over time^11^.

Teaching activities are also part of the tasks NACE-NUMETROP supervisors embrace, with UEPA-Santarém medical students and residents as target audiences. The center offers a monthly internship for medical residents from FMUSP, other nationally accredited institutions, and even foreign residents who are fluent in Portuguese. It also provides accommodation for participants. Applications from residents in several specialties (infectious diseases, family and community medicine, internal medicine, pediatrics, and clinical emergencies) are welcome and can be submitted to the corresponding author’s e-mail. 

Furthermore, NUMETROP develops community outreach activities that target health education, promotion, and prevention of locally prevalent diseases. NACE-NUMETROP encourages the internalization of physicians to the Amazon region, where specialized services are lacking not only in Family and Community Health, but also in other medical specialties^12^.

Academic outreach is meant to help medical residents to better understand local realities that differ from those seen in their original environment and promote dialogs leading to recognition of societal needs and respond accordingly. The initiative certainly contributes to citizenship development and fulfills the rights of vulnerable populations. Particularly in the Amazonian context, newly graduated medical health professionals may have this valuable experience enriched with knowledge exchange in Tropical Medicine, along with hands-on practice.

The idealizer and first coordinator of NACE/NUMETROP was the Full Professor Marcos Boulos (2001-2015), followed by Full Professor Aluísio Augusto Cotrim Segurado (2016 to April 2018). Since then this work has been coordinated by Associate Professor Francisco Oscar de Siqueira França and Associate Professor Ronaldo César Borges Gryschek. The most recent supervisory physicians at NUMETROP in Santarém are Alisson dos Santos Brandão, Helena Rangel Esper, João Guilherme Pontes Lima Assy, Kamila Vieira Silva, Olívia Campos Pinheiro, and Renato do Carmo Said.

For the last 20 years, NACE-NUMETROP has been working in the context of Brazilian public health, focusing on access to health services, diagnosis, and treatment of tropical and other infectious diseases. The results so far have been very rewarding for medical residents and supervisors, and the contributing faculty. It is, therefore, high time to celebrate. 
